# Age-Dependent Neuroplasticity Mechanisms in Alzheimer Tg2576 Mice Following Modulation of Brain Amyloid-β Levels

**DOI:** 10.1371/journal.pone.0058752

**Published:** 2013-03-15

**Authors:** Anna M. Lilja, Jennie Röjdner, Tamanna Mustafiz, Carina M. Thomé, Elisa Storelli, Daniel Gonzalez, Christina Unger-Lithner, Nigel H. Greig, Agneta Nordberg, Amelia Marutle

**Affiliations:** 1 Alzheimer Neurobiology Center, Department of Neurobiology, Care Sciences and Society, Karolinska Institutet, Stockholm, Sweden; 2 Department of Geriatric Medicine, Karolinska University Hospital Huddinge, Stockholm, Sweden; 3 Drug Design & Development Section, Laboratory of Neurosciences, Intramural Research Program, National Institute on Aging, National Institutes of Health, Baltimore, Maryland, United States of America; Oregon Health & Science University, United States of America

## Abstract

The objective of this study was to investigate the effects of modulating brain amyloid-β (Aβ) levels at different stages of amyloid pathology on synaptic function, inflammatory cell changes and hippocampal neurogenesis, i.e. processes perturbed in Alzheimer’s disease (AD). Young (4- to 6-month-old) and older (15- to 18-month-old) APP_SWE_ transgenic (Tg2576) mice were treated with the AD candidate drug (+)-phenserine for 16 consecutive days. We found significant reductions in insoluble Aβ1-42 levels in the cortices of both young and older transgenic mice, while significant reductions in soluble Aβ1-42 levels and insoluble Aβ1-40 levels were only found in animals aged 15–18 months. Autoradiography binding with the amyloid ligand Pittsburgh Compound B (^3^H-PIB) revealed a trend for reduced fibrillar Aβ deposition in the brains of older phenserine-treated Tg2576 mice. Phenserine treatment increased cortical synaptophysin levels in younger mice, while decreased interleukin-1β and increased monocyte chemoattractant protein-1 and tumor necrosis factor-alpha levels were detected in the cortices of older mice. The reduction in Aβ1-42 levels was associated with an increased number of bromodeoxyuridine-positive proliferating cells in the hippocampi of both young and older Tg2576 mice. To determine whether the increased cell proliferation was accompanied by increased neuronal production, the endogenous early neuronal marker doublecortin (DCX) was examined in the dentate gyrus (DG) using immunohistochemical detection. Although no changes in the total number of DCX^+^-expressing neurons were detected in the DG in Tg2576 mice at either age following (+)-phenserine treatment, dendritic arborization was increased in differentiating neurons in young Tg2576 mice. Collectively, these findings indicate that reducing Aβ1-42 levels in Tg2576 mice at an early pathological stage affects synaptic function by modulating the maturation and plasticity of newborn neurons in the brain. In contrast, lowering Aβ levels in Tg2576 mice when Aβ plaque pathology is prominent mainly alters the levels of proinflammatory cytokines and chemokines.

## Introduction

The accumulation of amyloid-β (Aβ) aggregates in the brain is a pathological hallmark of Alzheimer’s disease (AD). Aβ is thought to play a central role in the disease pathogenesis, triggering a cascade of neurodegenerative processes including the activation of inflammatory mediators, altered protein kinase and neurotrophic signaling, oxidative stress, and neuronal and synaptic dysfunction, ultimately resulting in the impairment of cognitive functions in AD patients [1], [2], [3], [4], [5].

Recent advances in molecular imaging have provided a better understanding of the time course of pathological changes in the brain during disease progression. *In vivo* positron emission tomography (PET) imaging with amyloid tracers such as Pittsburgh Compound B (^11^C-PIB), has demonstrated that increased fibrillar Aβ deposition in the brain precedes functional changes and cognitive decline in AD patients [6], [7], [8]. ^11^C-PIB PET imaging has also been used to measure changes in brain Aβ load in patients with mild AD treated with the potential therapeutical drug (−)-phenserine [9], a non-competitive acetylcholinesterase inhibitor with reported modulatory effects on Aβ production [10], [11]. A reduction in ^11^C-PIB retention was observed in some patients, and cerebrospinal fluid (CSF) Aβ40 levels correlated positively with improvement in brain metabolic function and cognition in AD patients [9].

(+)-Phenserine (also known as posiphen) is also under consideration for AD treatment. Unlike its enantiomer (−)-phenserine, this molecule provides little acetylcholinesterase inhibitory action; it lowers the generation of Aβ by suppressing amyloid precursor protein (APP) synthesis [12]. A recent pharmacokinetic study undertaken in a small group of patients with mild cognitive impairment who were treated with (+)-phenserine reported significantly lower levels of sAPPα, sAPPβ and proinflammatory markers in the CSF, and alterations in CSF Aβ42 levels [13].

Clinical and biomarker changes assessed in patients with a genetic predisposition for familial AD (FAD) suggest that pathological changes start approximately two decades before cognitive symptoms appear [14].

Transgenic mice harboring similar mutations in human APP and presenilin-1 (PS1) genes exhibit early and progressive accumulation of Aβ, associated with compromised neocortical synaptic plasticity and synaptic dysfunction, traits similar to those observed in FAD patients [15], [16], [17]. The complex composition of pathological alterations in the AD brain microenvironment is thought to lead to impairment of neurotrophic signaling and inadequate synaptic repair [18], [19], [20].

Neurogenesis, the birth of new neurons, has been shown to persist in the adult brain, although it is largely restricted to two regions: the subventricular zone of the lateral ventricles and the subgranular zone of the dentate gyrus (DG) in the hippocampus. However, the ability of new neurons to incorporate into the brain circuitry and form functional synaptic connections declines with age [21].

In the few existing neurogenesis studies performed in the autopsied brains of AD patients, an increase in the number of neural progenitors in the DG has been demonstrated, compared to age-matched controls [22], [23], [24]. Whether this rise in neural progenitors is a consequence of triggered compensatory mechanisms related to the neurodegenerative changes in the brain or is induced by medications the patients received before death remains unclear. Nevertheless, this interesting finding implies that stimulation of the neuroprotective and regenerative mechanisms in the AD brain is feasible and, hence, this area holds promise as a novel treatment approach for slowing AD progression.

Studies in various AD animal models have tested the potential of stimulating neurogenesis with different treatment approaches. We have previously demonstrated improved survival and *in vivo* neuronal differentiation of transplanted neural progenitors in the brains of APP23 transgenic mice receiving (+)-phenserine treatment before the onset of Aβ plaque deposition, compared with APP23 mice that did not receive treatment [25]. Other pharmacological strategies, including Aβ immunization or treatment with neurotrophic peptides, have also been associated with enhanced hippocampal neurogenesis in other AD transgenic mice strains [26], [27], [28].

A paradigm shift in recent years has resulted in the suggestion that anti-Aβ therapies, in combination with disease-modifying therapies, could be highly effective in preventing or delaying the development of the disease in asymptomatic patients with very early signs of AD pathology [29]. In line with this view, clinical studies focusing on early presymptomatic treatment and prevention are being initiated, as presented at the most recent meeting for clinical trials on AD (CTAD) (see http://www.alzforum.org/new/detail.asp?id=3312).

This preclinical study was designed to examine the effects on synaptic function and neuroinflammatory and regenerative processes of an Aβ-lowering therapeutic intervention at different stages of amyloid pathology in an AD transgenic mouse model. We treated transgenic mice overexpressing human APP with the Swedish double mutation (Tg2576), at 4–6 and 15–18 months of age, with (+)-phenserine for 16 days. These mice exhibit impaired cognitive functioning and high soluble Aβ levels in the brain when very young (<6 months old); insoluble Aβ levels are increased and Aβ plaques are formed later [30], [17].

We measured a selective decrease in Aβ42 levels in the cortices of young Tg2576 mice and demonstrated attenuation of both Aβ40 and Aβ42 levels after the onset of Aβ plaque pathology in the brains of older mice. These changes were assessed in relation to levels of brain-derived neurotrophic factor (BDNF), the presynaptic vesicle protein synaptophysin, pro-inflammatory cytokines [interleukin-1β (IL-1β), tumor necrosis factor-alpha (TNF-α) and chemokine monocyte chemoattractant protein-1 (MCP-1)], and hippocampal neurogenesis. Concurrent with the reduction in amyloid levels, phenserine treatment primarily elevated cortical synaptophysin levels and increased the maturation of newborn neurons in the DG of 4- to 6-month-old Tg2576 mice, while significant alterations in IL-1β and MCP-1 were detected in the cortices of 15- to 18-month-old mice.

## Materials and Methods

### Animals

Male mice expressing the APP Swedish mutation (APPSWE2576Kha; Tg2576) were bred in the Karolinska Institutet animal care facility by backcrossing with B6SJL (F1) females (Taconic). Their genotype was determined using polymerase chain reaction technology [31]. Wild type (wt) littermates served as control animals throughout the study. All mice were housed in enriched cages with a 12-hr light-dark cycle and *ad libitum* access to food and water.

### Ethics Statement

All animal experimental procedures were carried out in strict accordance with the guidelines for the Swedish National Board for Laboratory Animals, and the protocol was approved by the Regional Ethics and Animal Research Committee at the Karolinska Institute, Stockholm, Sweden (Permit Numbers: S43/07 and S53/10). All surgery was performed under ketamine/xylazine anesthesia, and all efforts were made to minimize animal suffering.

### Drug Treatment

Tg2576 mice at 4–6 months (n = 16) and 15–18 months (n = 11–13) of age and age-matched wt controls (n = 13–16) were divided into groups of 6–8 mice and injected once daily with intraperitoneal (i.p.) (+)-phenserine tartrate (25 mg/kg) or physiological saline solution for 16 consecutive days. On the final three days, all animals were co-administered the thymidine analog 5-bromo-2′-deoxyuridine (BrdU; 50 mg/kg per day i.p.) to measure ongoing cell proliferation. Within 12 hr of the final administration, the mice were anesthetized with a 1∶1 mixture of ketamine (100 mg/kg) and xylazine (20 mg/kg) and euthanized by transcardial perfusion with phosphate buffered saline (PBS). The brains were collected and divided into hemispheres. One hemisphere was stored at −80°C and used later in the biochemical assays. The other hemisphere was post-fixed with 4% paraformaldehyde (PFA; pH 7.4) and transferred to a sucrose cryoprotectant for 24 h at 4°C, after which it was frozen at −80°C. Sagittal 20 µm brain sections were subsequently cut and processed for autoradiography binding and immunohistochemical staining.

### Tissue Extractions

Dissected cortical brain tissue was processed individually and sequential extraction steps were performed to obtain membranous and soluble fractions for Western blot and sandwich enzyme-linked immunosorbent assay (ELISA) analyses. Briefly, tissues were homogenized in ice-cold tris-buffered saline (50 mM Tris HCl, 150 mM NaCl, 1 mM EDTA, 1 mM DDT) containing protease inhibitors (Sigma). The homogenates were centrifuged (420×g, 10 min, 4°C) and the supernatant collected and re-centrifuged (15,000×g, 60 min, 4°C). The supernatant (cytosolic fraction) was saved, and the remaining pellet was resuspended in five volumes RIPA buffer containing detergent (2% Triton X-100, 0.2% SDS), centrifuged (15,000×g, 20 min, 4°C) and the supernatant (membranous fraction) collected. Both the cytosolic and membranous fractions were stored at −80°C until use.

### Aβ Measurements

Tris-soluble and -insoluble (guanidine-extracted) Aβ1-40 and Aβ1-42 levels were quantified in cortical homogenates using commercial colorimetric ELISA kits (Signal Select™ Human Aβ1–40 and 1–42, BioSource International Inc., Camarillo, CA, USA) as previously described [32]. Levels of Aβ were expressed as pg/mg tissue.

^3^H-PIB autoradiography binding was carried out by pre-incubating triplicate sections from each animal for 15 min in PBS buffer (pH 7.4) containing 1% bovine serum albumin (BSA) and then incubating them with 1.5 nM ^3^H-PIB (specific activity 68 Ci/mmol, custom synthesis, GE Healthcare, Germany) in PBS containing 0.1% BSA for 1 hr, as described earlier [33]. Non-specific binding was determined by incubating adjacent sections with 1 µM unlabeled PIB. After washing in PBS, the sections were exposed along with calibrated tritium standards (American Radiolabeled Chemicals, Saint Louis, MO, USA) on Fuji BAS-TR2040 phosphor imaging plates (Science Imaging Scandinavia AB, Nacka, Sweden) for 3 days. The plates were processed with a Fujifilm BAS5000 phosphorimager (Fuji, Tokyo, Japan), and binding densities were analyzed using Multigauge software V3.0 (Fuji). The relative optical density calculated from the tritium standards was used to calculate binding values in fmol/mg tissue.

Aβ1-42 and 4G8 immunohistochemistry analysis was carried out on 4% PFA sagittal brain sections treated with formic acid and 0.3% H_2_O_2_ to deplete endogenous peroxidase activity. Following washing and blocking steps, the sections were incubated with antibodies Aβ1-42 and 4G8 (reactive to amino acid residue 17–24; 1∶500, Covance, Denver, PA) overnight at 4°C. The following day, the sections were rinsed and incubated for 1 hr with a biotinylated anti-mouse antibody IgG (1∶200; Vector laboratories Inc., Burlingame, CA). Immunoreactivity was visualized using the avidin biotinylated peroxidase complex (ABC) method (Vector Laboratories Inc., Burlingame, CA) with the sections counterstained with hematoxylin.

### Growth Factor and Cytokine Measurements

The levels of BDNF and the proinflammatory cytokines IL-1β, TNF-α and chemokine MCP-1 were measured in cortical cytosolic fractions using commercial ELISA kits, the mouse BDNF ELISA kit (Abnova, Immunkemi F&D AB, Stockholm, Sweden) and the MSD Multi-array® mouse cytokine ultra-sensitive assay (Meso Scale Discovery, Gaithersburg, MD, USA), according to the manufacturer’s instructions.

### Western Blot Detection of Synaptophysin Protein

Samples from the membranous fractions (15 µg protein) were subjected to electrophoresis in 12% Tris-Bis gels (Invitrogen) and transferred to polyvinylidene difluoride (PVDF) membranes (GE Healthcare). The membranes were blocked with 5% non-fat dry milk and incubated overnight at 4°C with rabbit anti-synaptophysin (1∶2000, DAKO), with rabbit anti-β-actin as the loading control (1∶2000, Abcam) and, thereafter, with a horseradish peroxidase (HRP)-conjugated donkey anti-rabbit secondary antibody (1∶2000, Santa Cruz Biotechnology) for 2 hr at 21°C. Protein bands were visualized using an enhanced chemiluminescence detection reagent (GE HealthCare) and the density of each band was normalized with β-actin and compared with a pooled sample using the National Institutes of Health Image J analysis software program.

### BrdU and Doublecortin (DCX) Immunohistochemistry

A one-in-six series of sections throughout the entire rostral-caudal extent of the hippocampus was used to assess the number of BrdU-positive (BrdU^+^) cells. BrdU fluorescent immunohistochemistry analysis was carried out by incubating the free-floating sections with sheep anti-BrdU (1∶100, Abcam, Cambridge, UK) overnight at 4°C and then with a Texas Red conjugated donkey anti-sheep IgG antibody (1∶500, Santa Cruz Biotechnology, Inc., Santa Cruz, CA) for 2 hr at 21°C. The sections were mounted with 4′, 6-diamidino-2-phenylindole (DAPI) for nuclear counterstaining (Vector Laboratories). The total number of BrdU^+^ cells in the hippocampus was quantified using a Nikon E800 Eclipse fluorescence microscope (Nikon, Tokyo, Japan) at 20X magnification. An average of three sections was counted for each animal and quantification was blinded.

For the detection of DCX-expressing cells, sections were incubated overnight with goat anti-DCX (1∶500, Santa Cruz) and the immunoreactivity was visualized with rabbit anti-goat streptavidin-horseradish peroxidase conjugate (1∶500, Vector Laboratories) using the ABC method with nickel-enhanced diaminobenzidine as substrate. Images were captured with a light microscope (Leica Microsystems, Wetzlar, Germany) with an attached ProgRes® video camera and the ProgRes®Capture Pro2.8.8 image analysis system (Jenoptik AG, Jena, Germany) was used at 20X magnification. The total number of DCX^+^ cells and the number of DCX^+^ cells with clearly defined dendrites extending from the cell body were counted in the DG in 4- to 6- and 15- to 18-month-old Tg2576 and wt mice (triplicate sections per animal; blinded analysis).

### Statistical Analyses

Data are expressed as mean values ± SEM. Statistical differences between groups were determined with the non-parametric Mann-Whitney test or the unpaired t-test using a statistical software package (GraphPad Prism, version 4.00; GraphPad Software, San Diego, CA). The non-parametric Spearman’s correlation was used to assess the relationships between the different parameters. The significance level was set at p<0.05.

## Results

### (+)-Phenserine Decreases Brain Aβ Levels in 4- to 6- and 15- to 18-month-old Tg2576 Mice

**Figure 1:**
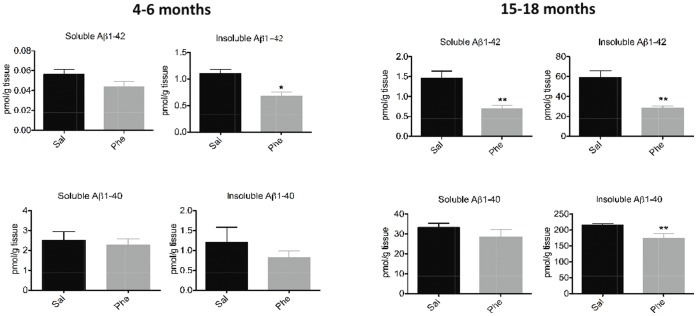
Effects of (+)-phenserine on brain amyloid levels in Tg2576 mice. Levels of tris-soluble (soluble) and guanidine-soluble (insoluble) Aβ1-42 and Aβ1-40 in the cerebral cortices of 4- to 6-month-old (**A**) and 15- to 18-month-old (**B**) Tg2576 transgenic mice treated with saline (Sal) or (+)-phenserine (Phe) for 16 days. * *P*<0.05 and **P<0.01 compared to saline-treated mice (Mann-Whitney test). Values are expressed as means ± SEM throughout the manuscript.

A significant decline (38%) in insoluble (guanidine-soluble) Aβ1-42 levels was measured in the cortices of 4- to 6-month-old (+)-phenserine-treated Tg2576 mice (*p*<0.05 vs saline-treated controls; Fig. 1A). In 15- to 18-month-old mice, both soluble and insoluble Aβ1-42 levels were significantly reduced (by 53% and 52%, respectively). A 20% decline in insoluble Aβ1-40 levels was also observed in (+)-phenserine-treated versus saline-treated older Tg2576 mice (*p*<0.01, Fig. 1B).

**Figure 2:**
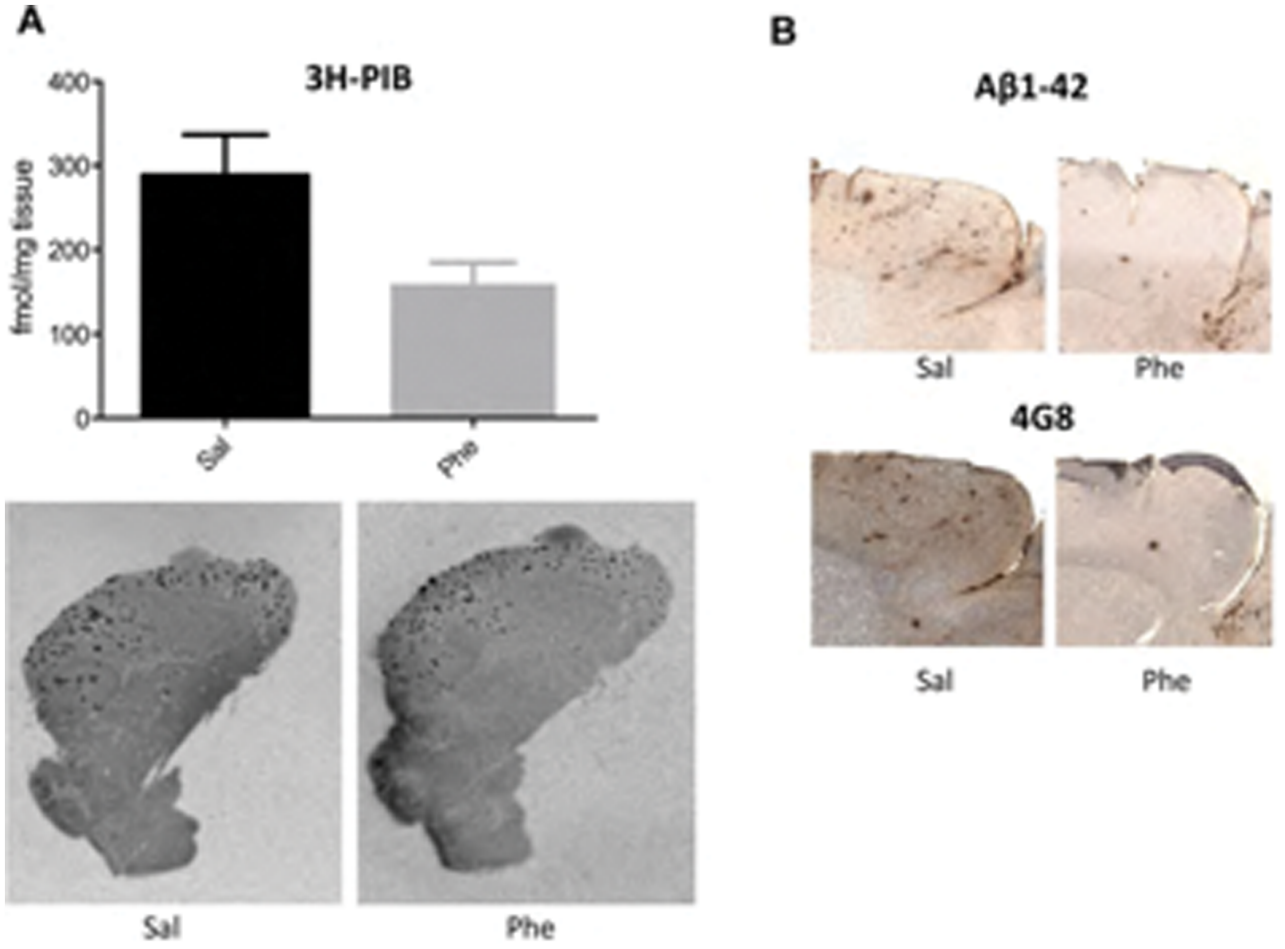
Modulation of amyloid-β (Aβ) burden in 15- to 18-month-old Tg2576 mice treated with (+)-phenserine. (**A**) *top* Binding of the amyloid ligand ^3^H-PIB (1.5 nM) to fibrillar Aβ in the brains of 15- to 18-month-old Tg2576 mice treated with saline or (+)-phenserine (Phe). A decrease in ^3^H-PIB binding was observed following (+)-phenserine treatment compared to saline treated mice (*p* = 0.05; Mann-Whitney test). *Bottom,* representative autoradiography distributions of ^3^H-PIB in sagittal hemibrain sections of saline- and (+)-phenserine-treated Tg2576 mice. (**B**) Representative sections illustrating immunohistochemical staining of amyloid plaques with antibodies Aβ1-42 and 4G8 in the cerebral cortices of 15- to 18-month-old Tg2576 mice treated with (+)-phenserine and saline.

Autoradiography with the PET ligand ^3^H-PIB was used to quantify the effects of (+)-phenserine on fibrillar Aβ deposition in brain sections from 15- to 18-month-old Tg2576 mice. At this age, these mice normally have pronounced fibrillar Aβ deposition in the brain. Reduced binding to fibrillar Aβ was observed in the cortical regions of (+)-phenserine-treated versus saline-treated mice (p = 0.05, Fig. 2A). Moreover, as illustrated in Figure 2B, Aβ1-42 and 4G8 immunoreactivity appeared weaker in the cerebral cortices of 15- to 18-month-old Tg2576 mice treated with (+)-phenserine versus saline treatment.

### Synaptophysin and BDNF Levels in the Cortices of 4- to 6- and 15- to 18-month-old Tg2576 Mice Treated with (+)-phenserine

**Figure 3:**
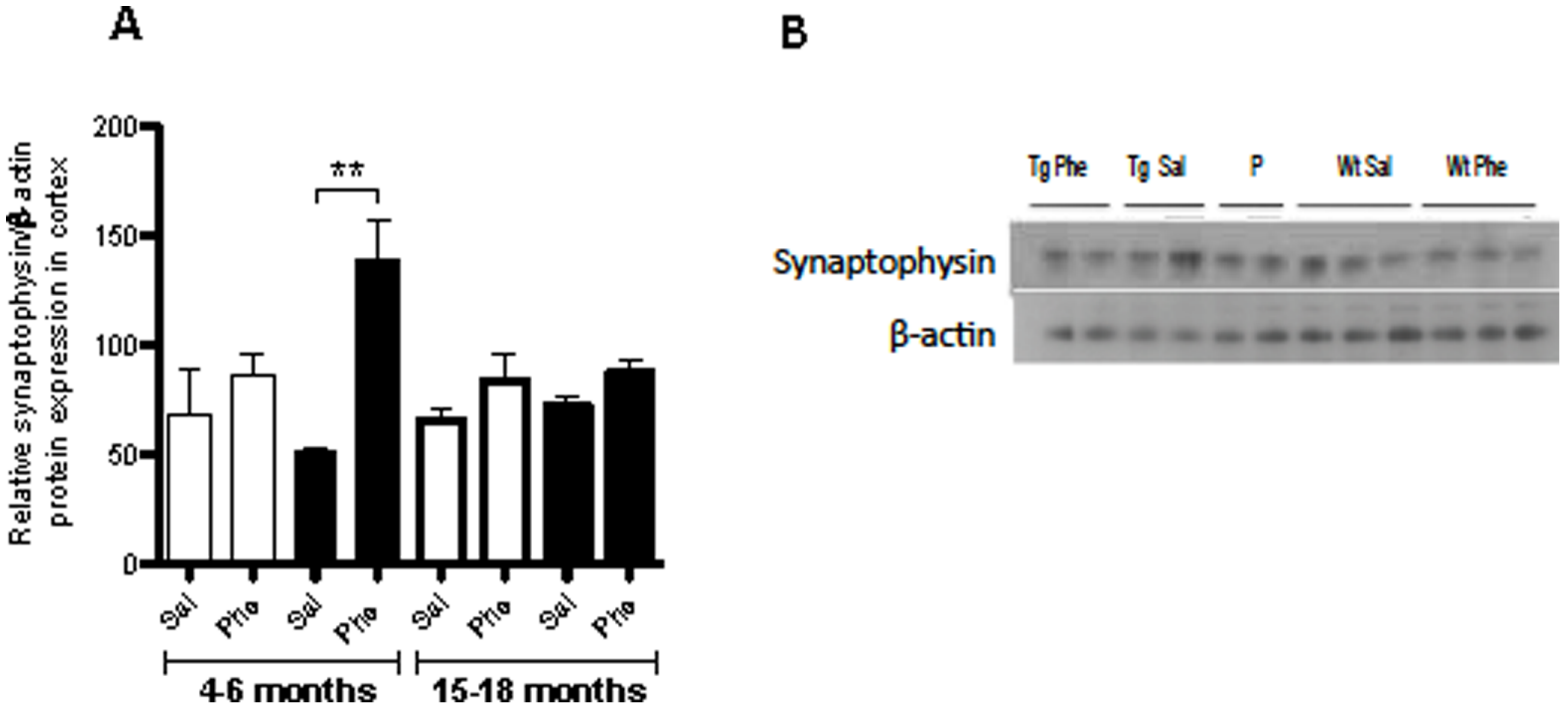
Synaptophysin protein levels in the cerebral cortices of 4- to 6- and 15- to 18-month-old wild-type (Wt, white bars) and Tg2576 mice (black bars) after treatment with saline (Sal) or (+)-phenserine (Phe). The signals corresponding to the synaptophysin levels were normalized to β-actin in each gel, and a pooled sample (P) was used to control the intergel variability. **P<0.01 compared to saline-treated 4- to 6-month-old Tg2576 mice (Mann-Whitney test). Values are expressed as means ± SEM.

To elucidate the effects of treatment on processes associated with synaptic plasticity, the expression of the presynaptic vesicle protein synaptophysin and neurotrophic BDNF protein was quantified in the cerebral cortices of Tg2576 mice at 4–6 and 15–18 months of age. Synaptophysin levels were significantly higher in the cerebral cortices of 4- to 6-month-old Tg2576 mice treated with (+)-phenserine than in those administered saline (*p*<0.01; Fig. 3). There were no significant differences in synaptophysin levels between the older Tg2576 mice and wt controls, or between (+)-phenserine and saline treatment in the older mice.

A significant elevation in BDNF levels was evident in older versus young Tg2576 mice (3626±81 versus 867±129 pg/mg protein; p<0.01). A trend towards increased BDNF levels was observed in the drug-treated young and older Tg2576 and wt mice versus saline treatment, but this did not reach statistical significance (data not shown).

### (+)-Phenserine Treatment Alters the Levels of Proinflammatory Cytokines IL-1β, TNF-α and Chemokine MCP-1 in the Brains of 15- to 18-month-old Tg2576 Mice

**Figure 4:**
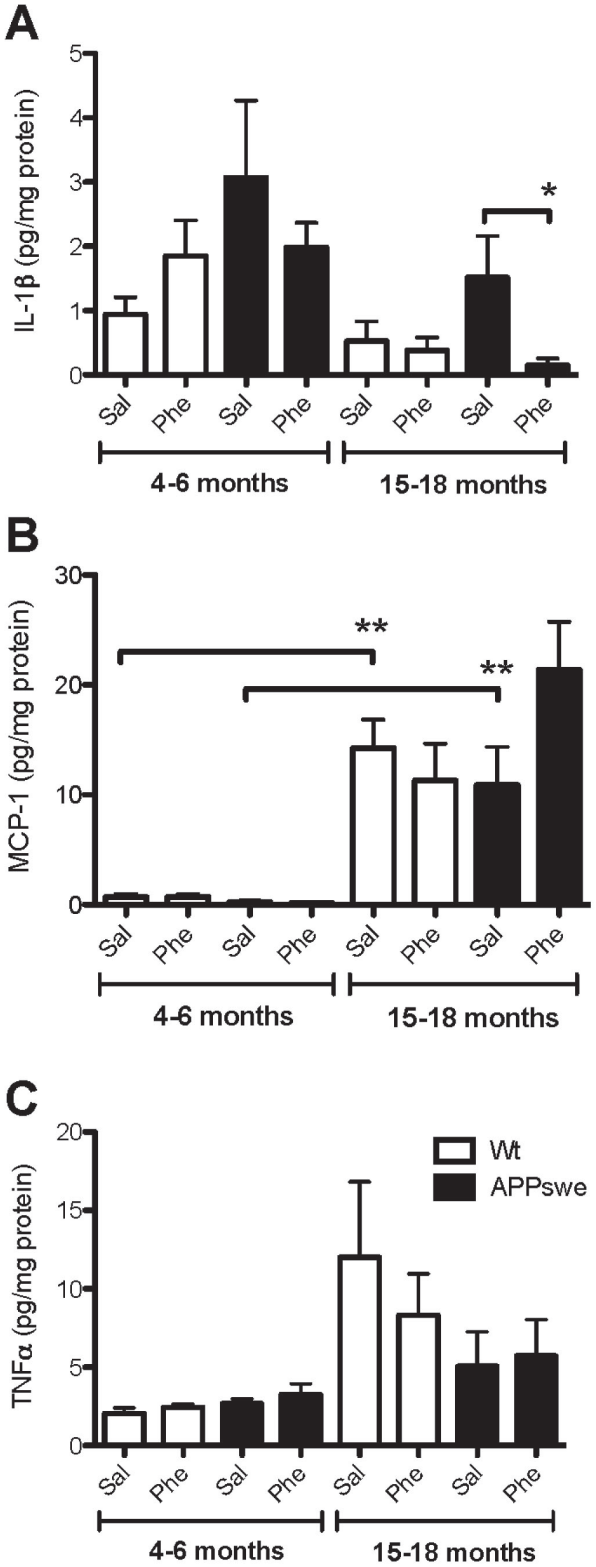
(+)-Phenserine-induced effects on proinflammatory cytokines (IL-1β, TNF-α) and a chemokine (MCP-1) in Tg2576 and wt mice brains. Effects of (+)-phenserine treatment on IL-1β levels (**A**), MCP-1 levels (**B**), and TNF-α levels (**C**) in the cerebral cortices of 4- to 6- and 15- to 18-month-old wild-type (Wt, white bars) and Tg2576 mice (black bars) treated with saline (Sal) or (+)-phenserine (Phe). *P<0.05 compared to saline-treated 15- to 18-month-old Tg2576 mice (Mann-Whitney test), **p<0.01 compared to saline-treated 4- to 6-month-old wildtype and Tg2576 mice (Mann-Whitney test). Values are expressed as means ±SEM.

A highly sensitive sandwich ELISA assay was used to examine whether the reduced cortical Aβ levels in the brains of young and older Tg2576 mice altered levels of the proinflammatory cytokines IL-1β, TNF-α and chemokine MCP-1. A significant decrease in IL-1β levels was observed in (+)-phenserine-treated Tg2576 mice at 15–18 months, but not at 4–6 months of age (Fig. 4A). Irrespective of treatment, MCP-1 levels were much lower in young Tg2576 and wt mice than in older animals (p<0.01; Fig. 4B). In the 15- to 18-month-old group of Tg2576 mice, MCP-1 levels increased after treatment but the difference did not reach statistical significance (Fig. 4B). In 4- to 6-month-old mice, there were no differences in TNF-α levels between drug- and saline-treated Tg2576 or wt mice, and the levels of TNF-α were lower than in older animals (Fig. 4C). At 15–18 months of age, we observed a trend for decreased TNF-α levels in (+)-phenserine-treated wt mice and increased TNF-α levels in (+)-phenserine-treated Tg2576 mice versus saline-treated mice (Fig. 4C).

### (+)-Phenserine Induces Age-dependent Increases in the Number of Proliferating Cells and Stimulates Dendritic Arborization of Newborn Neurons in the Hippocampi of Tg2576 Mice

**Figure 5:**
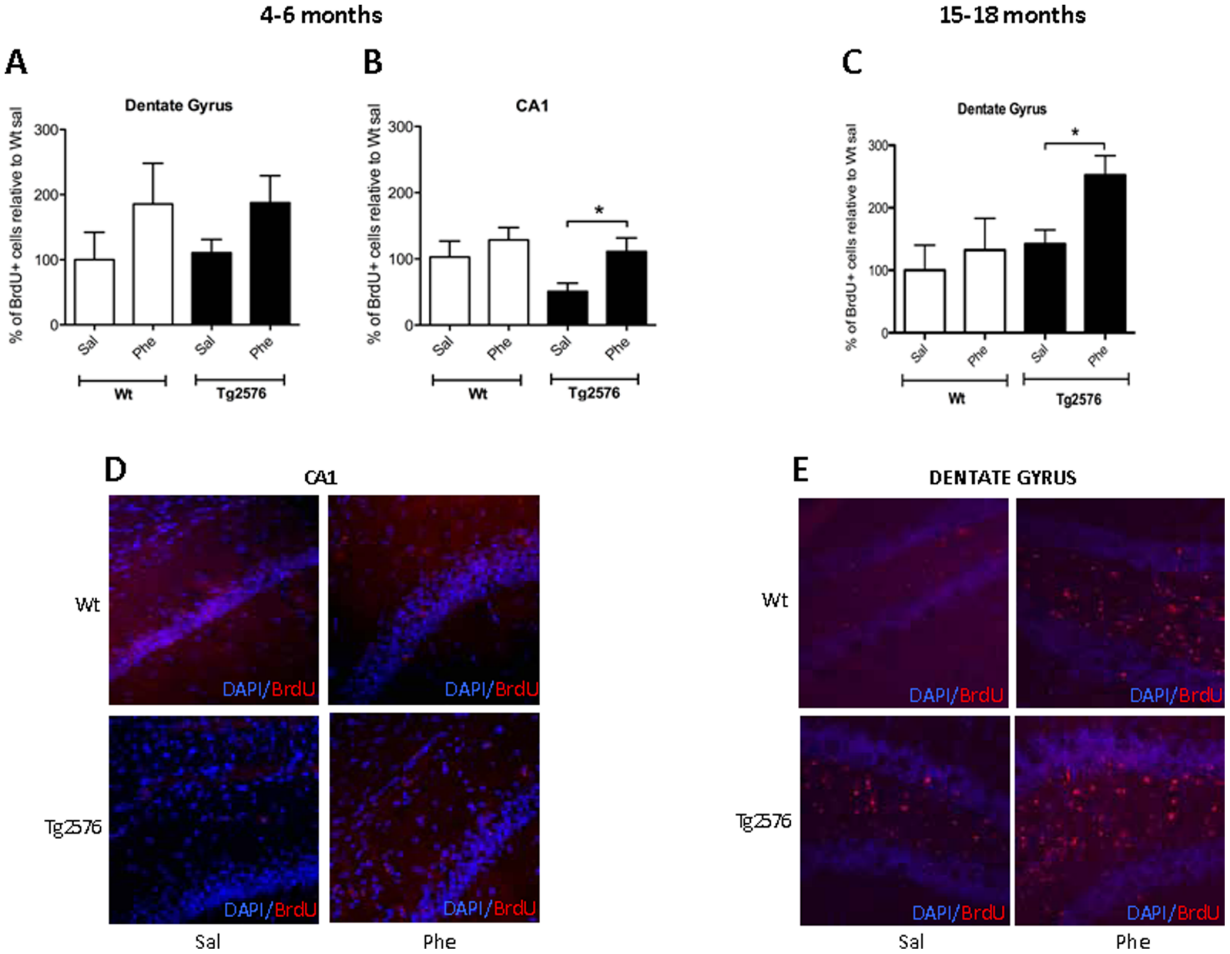
The effects of (+)-phenserine treatment on cell proliferation in the hippocampi of Tg2576 mice. The number of bromodeoxyuridine-positive (BrdU^+^) cells was increased in hippocampal regions of 4- to 6-month-old Tg2576 mice treated with saline (Sal) or (+)-phenserine (Phe). The number of BrdU^+^ cells was counted in the dentate gyrus (DG) (**A**), and in the CA1 region (**B**) in the hippocampi of wild-type (Wt) and Tg2576 mice. Representative images of the DG and CA1 in Tg2576 mice treated with saline (Sal) or (+)-phenserine (Phe) are shown in (**C**). The number of BrdU^+^ cells in the DG of 15- to 18-month-old Wt and Tg2576 mice treated with saline or (+)-phenserine is shown in Figures (**D**) and (**E**). *P<0.05 compared to saline-treated Tg2576 mice aged 4–6 or 15–18 months (unpaired t-test). Values are expressed as means ± SEM. Sections were immunolabeled with BrdU (red) and the nuclei were stained with 4′,6-diamidino-2-phenylindole (DAPI, blue) and visualized at 20X magnification.

We then assessed both BrdU^+^ immunoreactivity (a marker for cell proliferation) and the expression of DCX (an endogenous neurogenesis marker for the microtubule-associated phosphoprotein that is expressed by migrating and differentiating neurons) in hippocampal sub-regions to see whether lowered Aβ levels stimulated regenerative mechanisms in the brains of Tg2576 mice at 4–6 and 15–18 months of age. At 4–6 months, the trend toward a greater number of BrdU^+^ cells observed in the DG (69% increase; Fig. 5A) reached significance in the CA1 region (119% increase, *p*<0.05; Fig. 5B and 5D) in Tg2576 mice treated with (+)-phenserine versus saline-treated mice. At age 15–18 months, the BrdU^+^ cell number was significantly elevated in the DG of Tg2576 (+)-phenserine-treated mice versus saline treatment (76% increase, *p*<0.05; Fig. 5C, E).

**Figure 6:**
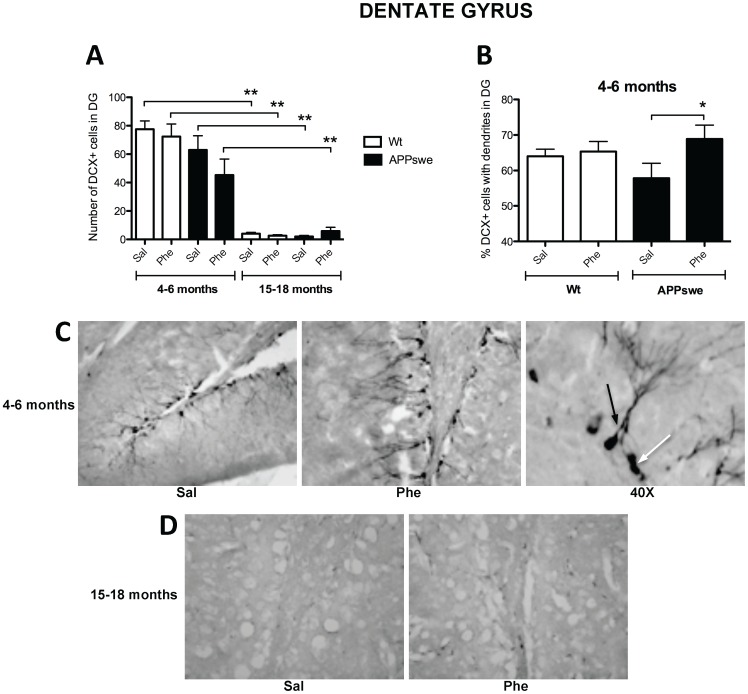
Increased dendritic arborization of newborn neurons in the dentate gyrus (DG) following treatment with (+)-phenserine. The number of doublecortin (DCX)-positive cells in the DG of 4- to 6- and 15- to 18-month-old mice after saline (Sal) or (+)-phenserine (Phe) treatedment (**A**), and the number of DCX^+^ cells with dendrites in 4- to 6-month-old mice (**B**). Illustrative images (20X magnification) of DCX immunoreactivity in 4- to 6-month-old Tg2576 mice that had been given saline or (+)-phenserine, and a 40X magnification of DCX^+^ cells with (black arrow) and without (white arrow) dendrites (**C**). Illustrative images (20X magnification) of DCX immunoreactivity in 15- to 18-month-old saline-treated and (+)-phenserine-treated Tg2576 mice (**D**). *P<0.05 compared to saline-treated 4- to 6-month-old Tg2576 mice, **p<0.01 compared to 4- to 6-month-old mice (Mann-Whitney test). Values are expressed as means ±SEM.

Immunohistochemical staining revealed the presence of DCX^+^ neurons in the granule cell layer and subgranular zone of the DG in both Tg2576 and wt mice. DCX^+^ cells were not detected in other hippocampal sub-regions. A marked reduction in DCX immunoreactivity was evident in the DG of Tg2576 mice at 15–18 months of age compared to those aged 4–6 months (*p*<0.05, Fig. 6A, C and D). However, the total number of DCX^+^ neurons in the DG of mice in both age groups was unaffected by (+)-phenserine treatment. After further quantification of DCX^+^ neurons, those possessing dendrites were determined to be significantly elevated in number in the DG of 4- to 6-month-old Tg2576 (+)-phenserine-treated mice versus those receiving saline (*p*<0.05, Fig. 6B).

### Aβ Reduction is Associated with Age-related Effects on Cell Proliferation, Synaptogenesis and Inflammatory Cytokines and Chemokines in Tg2576 Mice

**Figure 7:**
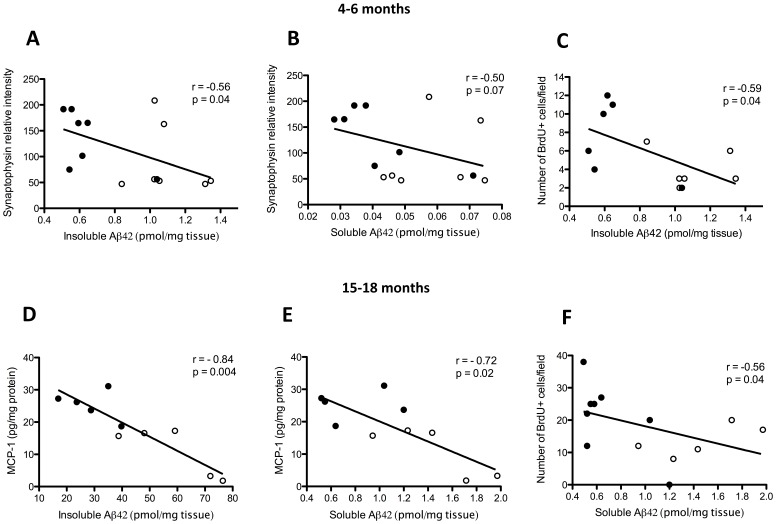
Correlations of amyloid-β (Aβ) levels with synaptophysin levels, cell proliferation and proinflammatory markers in the brains of 4- to 6- and 15- to 18-month-old Tg2576 mice. Correlation of synaptophysin levels with cortical guanidine-soluble (insoluble) Aβ1-42 levels (**A**) and tris-soluble (soluble) Aβ1-42 levels (**B**). Correlation of insoluble Aβ1-42 levels with the number of bromodeoxyuridine-positive (BrdU^+^) cells (**C**) in the dentate gyri of 4- to 6-month-old Tg2576 mice. In Tg2576 mice aged 15–18 months, the relationships between MCP-1 levels and insoluble and soluble Aβ1-42 levels in the cortex are shown in (**D)** and (**E**), respectively, and the relationship between the number of proliferating BrdU^+^ cells in the dentate gyrus and cortical soluble Aβ1-42 levels is shown in (**F**) (Spearman rank test). Open circles indicate saline-treated animals (n = 5–7) and closed circles indicate (+)-phenserine-treated animals (n = 5–8).

The relationship between Aβ levels and the expression of synaptophysin, BDNF, and proinflammatory markers was examined in the brains of both young and older Tg2576 mice. At 4–6 months of age, a significant inverse correlation was observed between insoluble Aβ1-42 levels and synaptophysin protein levels (p<0.05; Fig. 7A), and a weaker inverse correlation was seen between soluble Aβ1-42 levels and synaptophysin levels (p = 0.07; Fig. 7B). The levels of insoluble Aβ1-42 correlated negatively with the number of BrdU^+^ cells in the DG (p<0.05; Fig. 7C). No correlation, however, was apparent between Aβ1-42 levels and DCX^+^-expressing cells (data not shown). In 15- to 18-month-old Tg2576 mice, levels of MCP-1 but not of the cytokines IL-1β and TNF-α demonstrated a significant negative correlation with both insoluble (p<0.01; Fig. 7D) and soluble (p<0.05; Fig. 7E) Aβ1-42 levels. Similarly, the number of BrdU^+^ cells in the DG was inversely correlated with cortical soluble Aβ1-42 levels (p<0.05) but not insoluble Aβ1-42 levels (Fig. 7F).

## Discussion

The aim of this study was to elucidate the extent to which pharmacological modulation of brain Aβ levels at different stages of amyloid pathology influences synaptic, neuroinflammatory and regenerative processes in the brains of AD Tg2576 mice.

These mice accumulate Aβ in an age-related manner, particularly in the cortex and the hippocampus, and although overt neuronal loss is not observed with ageing, synaptic aberrations and progressive learning and memory deficits arise. These are attributed to the presence of soluble Aβ assemblies at younger ages and, at later stages, with heavy Aβ plaque deposits [34], [30]. We report here a pronounced reduction in Aβ, primarily Aβ1-42, levels in the cortices of 4- to 6- and 15- to 18-month-old Tg2576 mice following treatment with (+)-phenserine. This drug is currently undergoing clinical optimization and evaluation for AD therapy [13]. Decreases in insoluble Aβ1-42 levels detected by ELISA were more prominent in 15- to 18-month-old Tg2576 mice than in younger mice, in accord with the higher rate of Aβ plaque deposition with age. These decreases were supported by reduced Aβ42 immunohistochemistry and autoradiographic distribution of ^3^H-PIB, which binds to fibrillar Aβ. The finding that (+)-phenserine has a greater impact on Aβ1-42 than on Aβ1-40 levels is in agreement with a prior preclinical study [12].

The neurodegenerative process in AD is initially characterized by synaptic damage accompanied by neuronal loss. Synaptic dysfunction is one of the strongest correlates to cognitive impairment in patients with AD [35]. Repair mechanisms in the brain can be associated with neuroplasticity at multiple levels, including structural remodeling of degenerating neuronal circuits, synaptic changes and strengthening of the dendritic branches that connect synapses. In this study, elevated levels of the synaptic protein synaptophysin were seen in the cortices of 4- to 6-month-old (+)-phenserine-treated Tg2576 mice, supporting the premise that lowering brain Aβ levels early in the disease could have beneficial effects on synaptogenesis.

The neurotrophic factor BDNF plays an important role in promoting neuronal survival and synaptic plasticity [36], [37]. The levels of BDNF are decreased in both the cerebral cortex and the hippocampus in the AD brain, and it is thought that the early impairments in synaptic function could result in part from neurotrophin signaling deficits [18], [19].

A previous study in AD Tg2576 mice demonstrated that Aβ reduces BDNF signaling by impairing axonal transport of BDNF [38] while another study reported that engrafted neural stem cells expressing high levels of BDNF could improve the spatial learning and memory deficits observed in aged 3×Tg AD mice [39]. In our study, only slight increases in BDNF brain levels were measured in young and older Tg2576 mice treated with (+)-phenserine. Interestingly, we found that BDNF levels were significantly higher in the older versus younger saline treated Tg2576 cohorts, suggesting an age-associated compensatory up-regulation of brain BDNF due to increasing Aβ in the brain. Thus, the regulation of BDNF expression as well as signaling in AD brain appears to be complex, and further studies are warranted to fully characterize (+)-phenserine’s actions on BDNF signaling and also that of other neurotrophins during the course of AD neurodegeneration.

An inflammatory response invariably accompanies elevated Aβ levels in the brain, with a hallmark of increased presence of activated microglia and reactive astrocytes in the brains of AD patients [40], [41]. Compelling evidence supports a key role for microglia and astrocytes in regulating and maintaining neuronal activity, which can be adversely influenced by elevated Aβ levels [42]. Aβ increases the synthesis of microglia and reactive astrocytes and the release of pro-inflammatory cytokines [43], [44], [45], as well as inhibiting adult neurogenesis [46], [47], [48]. Our study showed elevated brain IL-1β levels in both 4- to 6- and 15- to 18-month-old Tg2576 mice, signifying early and persistent activation of inflammatory processes. (+)-Phenserine reduced IL-1β levels in the older cohort in comparison to those measured in wt littermates. IL-1β has been shown to decrease synaptophysin levels in cortical neuronal cultures, and to inhibit cell proliferation in the DG of rodents [49]. However, the changes in IL-1β levels that we measured did not correlate with either synaptophysin levels or with increased cell proliferation.

An age-related rise in MCP-1 levels was also detected in the cortices of aged wt and Tg2576 mice. MCP-1 induces astrocyte chemotaxis and contributes to the recruitment of astrocytes around Aβ plaques [50]. It has recently been suggested that astrocytes play an additional role as key integrators of neurogenic permissiveness [51]. In neurogenic brain regions, new astrocytes are produced alongside new neurons [52], [53].

In the present study, we can not dismiss the possibility that the increase in cell proliferation observed in the hippocampi following Aβ reduction may have contributed to a rise in astroglia precursors. Since BrdU was administered to the mice only during the last three days following (+)-phenserine treatment, co-staining BrdU^+^ cells with an astrocytic marker for assessment of non-neuronal identity was not applicable under the experimental conditions in this study. The greatest increase in MCP-1 levels was observed in 15- to 18-month-old Tg2576 (+)-phenserine-treated mice, and a reduction in Aβ42 levels was associated with a rise in MCP-1 levels only in older mice, which suggests induction of astrocyte-mediated neuroprotective effects. A similar elevation in TNF-α levels was observed in older wt and Tg2576 mice compared to the younger group. In addition, 15- to 18-month-old Tg2576 mice treated with (+)-phenserine demonstrated a tendency for increased TNF-α levels versus saline treatment but, because of variations among the mice in this group, this difference did not reach statistical significance. Both experimental and clinical evidence implicates the involvement of TNF-α in the pathogenesis of AD [43], [54], [55]. However, there are also a few studies that report a neuroprotective function for TNF-α and a role in modulating neuronal cell function through an indirect mechanism by which TNF-α stimulates the production of neurotrophic factors [56], [57]. In support of the latter scenario, our results suggest the possibility that (+)-phenserine potentiates the neuroprotective function of TNF-α, as elevated levels of this cytokine were measured in the drug-treated 15- to 18-month-old mice, concomitant with high BDNF levels.

Neurogenesis plays an important role in structural neuronal plasticity and network maintenance in the adult brain. It is a complex developmental process characterized by five stages, in which neural stem/progenitor cells in the subgranular layer of the DG develop through proliferation, differentiation, migration, axonal and dendritic targeting, and finally functional synaptic integration into neuronal circuits [36], [23]. Previous studies in nonhuman primates and rodents have shown that immature neurons in the adult DG have high synaptic plasticity, and that this declines with age [58]. Critical for learning and memory, the hippocampus is one of the earliest regions to be affected in AD [59] and dysfunctional neurogenesis consequent to subtle early disease manifestations in the brain could in turn render neurons more vulnerable to AD and contribute to memory impairment [58], whereas enhanced neurogenesis could provide a compensatory, endogenous repair mechanism. Prior investigations into hippocampal neurogenesis in mouse models of AD have provided conflicting findings. The majority of these studies report compromised neurogenesis [60], [61], [62], but others have described increased neurogenesis [63], [64]. These contradictory findings may stem from the different transgenic models studied, the age of the mice, or the detection methods used to label proliferating cells.

In our study, (+)-phenserine treatment of young adult Tg2576 mice resulted in significantly increased numbers of BrdU^+^ proliferating cells in the CA1 region, an area particularly vulnerable to Aβ deposition [65], and a trend for increased numbers in the DG. A similar increase in BrdU^+^ proliferating cells was observed in the DG of older Tg2576 mice following treatment. DCX is expressed in transiently amplifying neuroblasts during the migration and early differentiation phase of neurogenesis. DCX expression was therefore quantified to determine whether the increase in the number of BrdU^+^ proliferating cells in the DG was accompanied by an increase in neuronal production. No net rise in DCX^+^ cells was apparent in the DG of 4- to 6-month-old Tg2576 mice, but increased dendritic arborization was seen in differentiating neurons in this region following (+)-phenserine treatment. Markedly fewer DCX^+^ cells were identified in the DG of both saline- and drug-treated 15- to 18-month-old Tg2576 mice, indicating that neural stem/progenitor cells are more vulnerable in older animals.

In conclusion, the findings reported in this study suggest that early modulation of Aβ levels with (+)-phenserine could influence the maturation and plasticity of newborn neurons in the brain. In addition, reducing the Aβ load in the AD brain when Aβ plaque pathology is prominent could support neuroprotective functions by altering the levels of proinflammatory cytokines and chemokines.

In light of current discussions regarding anti-Aβ treatment in clinical trials aiming to promote effective Aβ reduction or clearance in presymptomatic patients and patients with mild AD, it is important to continue carrying out translational studies in preclinical AD transgenic animal models to test the long-term effects of Aβ-lowering drugs and to elucidate whether targeting Aβ pathways early in the disease will lead to positive effects on brain function, including the stimulation of brain repair.
