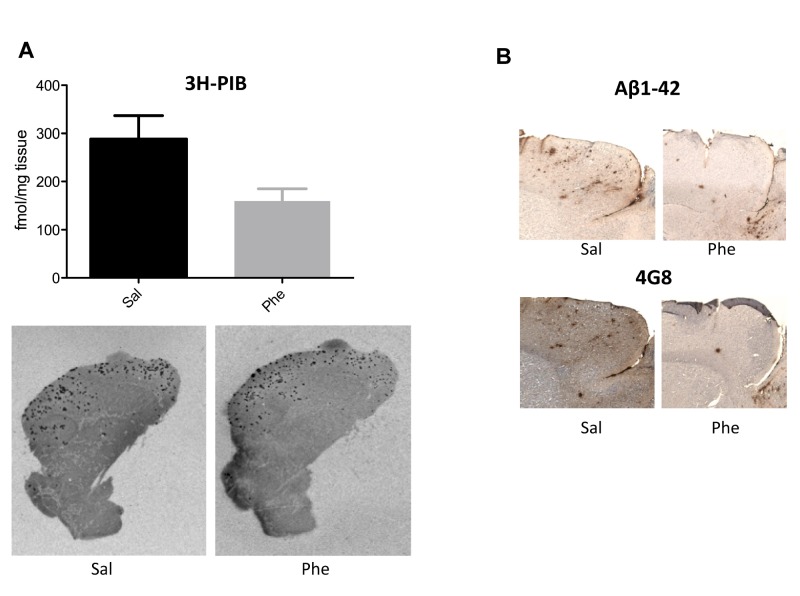# Correction: Age-Dependent Neuroplasticity Mechanisms in Alzheimer Tg2576 Mice Following Modulation of Brain Amyloid-β Levels

**DOI:** 10.1371/annotation/79c850e6-4494-4533-b68d-ef4eb72cd65a

**Published:** 2013-10-10

**Authors:** Anna M. Lilja, Jennie Röjdner, Tamanna Mustafiz, Carina M. Thomé, Elisa Storelli, Daniel Gonzalez, Christina Unger-Lithner, Nigel H. Greig, Agneta Nordberg, Amelia Marutle

An error occurred during the production process affecting the readability of Figure 2. A more clear Figure 2 can be found here: 

**Figure:**